# Supplementary material for: Content-rich biological network constructed by mining PubMed abstracts
Source: BMC Bioinformatics. 2004 Oct 8;5:147. doi: 10.1186/1471-2105-5-147 (PMC528731; doi:10.1186/1471-2105-5-147)
Supplement: Additional File 2 — The original results of the above study (non-essential files are deleted to keep the file size under the limit set by BMC bioinformatics). [file 1471-2105-5-147-S2.bz2 › chilibotAdditionalFile2/dip50/723ID9180258E18063/html/SECG_SECY.html]

 


 **SECG** and **SECY** 
  
Found 41 abstracts in PubMed, retrieved 41.  
 

 What does Google say? 
 PDF only 
| .edu only 

---

**Interactive relationship** (e.g. stimulation, inhibition, etc)

**Neutral relationship**- **SecG**  overproduction also stabilized the overproduction of  **SecY** .  Ref: 8526914 Biochem Biophys Res Commun, 1995
- These results collectively suggest that a C2 TM3 P2 TM4 C3 region of  **SecY**  serves as an interface with  **SecG** .  Ref: 12809499 Biochemistry, 2003
- His6  **SecY**  brought down both SecE and  **SecG** , as expected.  Ref: 9180258 FEBS Lett, 1997
- By analogy with  **secY**  prlA, secA prlD, and secE prlG,  **secG**  could therefore be called  **secG**  prlH.  Ref: 9020118 J Biol Chem, 1997
- Likewise,  **SecY**  SecE complex could be isolated from the  **secG**  deleted strain.  Ref: 9180258 FEBS Lett, 1997
- These results indicate that  **SecG**  preferentially interacts with SecE, which associates with  **SecY** , the  **SecG**  SecE  **SecY**  complex thus being formed in the cytoplasmic membrane.  Ref: 8526914 Biochem Biophys Res Commun, 1995
- The E. coli enzyme is required for proteolytic degradation of some unstable proteins that include both soluble regulatory proteins such as sigma 32 heat shock sigma factor and phage lambda CII transcriptional activator, and membrane proteins including uncomplexed forms of  **SecY**  forms the translocon together with SecE and  **SecG**  and the a subunit of the F0 complex of the H ATPase.  Ref: 10077851 FEMS Microbiol Rev, 1999
- Thus, the C2 C3 region of  **SecY**  is in the proximity of the N terminal half of the  **SecG**  cytoplasmic loop.  Ref: 12809499 Biochemistry, 2003
- These  **SecY**  Cys residues also formed a disulfide bond with an engineered cytosolic cysteine at position 28 of  **SecG** .  Ref: 12809499 Biochemistry, 2003
- Whereas homologues of Sec61alpha  **SecY**  and Sec61gamma SecE exist in archaea, identification of the third component Sec61beta or  **SecG**  has remained elusive.  Ref: 11943537 Trends Biochem Sci, 2002
- Overproduction of  **SecG**  stabilized the overproduction of SecE C, an unstable truncated derivative of SecE, as effectively as  **SecY**  does.  Ref: 8526914 Biochem Biophys Res Commun, 1995
- From the extent of  **SecG**  overproduction, the number of  **SecG**  molecules in one normal cell was estimated to be about 00, which is similar to those of  **SecY**  and SecE.  Ref: 8526914 Biochem Biophys Res Commun, 1995
- In addition, mutations in  **secG**  were isolated as suppressors against a cold sensitive mutation secY104 affecting the TM4 C3 boundary of  **SecY** .  Ref: 12809499 Biochemistry, 2003
- Taken together, these results indicate that  **SecG**  enhances the translocation activity of the apparatus after the step of SecA targeting to  **SecY** .SecE.  Ref: 8089132 J Biol Chem, 1994
- To facilitate large scale purification of the SecYEG heterotrimer,  **SecY**  was fused at its amino terminus with a hexahistidine tag and co overexpressed with SecE and  **SecG** .  Ref: 9425040 Biochemistry, 1998

**Non-interactive relationship** (e.g. studied together, co-existance, homology, etc.)

- Protein export in Escherichia coli is mediated by translocase, a multisubunit membrane protein complex with SecA as the peripheral subunit and the  **SecY** , SecE, and  **SecG**  proteins as the integral membrane domain.  Ref: 10074070 J Bacteriol, 1999
- In the bacterial cytoplasmic membrane, the channel is formed by oligomers of a heterotrimeric membrane protein complex consisting of subunits  **SecY** , SecE and  **SecG** .  Ref: 12167867 Nature, 2002
- This SRP, assisted by its receptor, FtsY, mediates the transfer to a common translocation site in the inner membrane that contains SecA,  **SecG** , and  **SecY** .  Ref: 10514469 J Biol Chem, 1999
- i Formaldehyde cross linking of translocase reveals cross links between  **SecY** , SecE and  **SecG** , but not higher order oligomers.  Ref: 10944122 EMBO J, 2000
- Escherichia coli preprotein translocase comprises a membrane embedded hexameric complex of  **SecY** , SecE,  **SecG** , SecD, SecF and YajC SecYEGDFyajC and the peripheral ATPase SecA.  Ref: 9305629 EMBO J, 1997
- The transport of large preproteins across the Escherichia coli plasma membrane is catalyzed by preprotein translocase, comprised of the peripherally bound SecA subunit and an integrally bound heterotrimeric domain consisting of the  **SecY** , SecE, and  **SecG**  subunits.  Ref: 7650029 J Biol Chem, 1995
- E. coli SecA, but not B. subtilis SecA, supported efficient ATP dependent translocation of the E. coli precursor OmpA proOmpA into inner membrane vesicles containing the hybrid SecYEG complexes, if E. coli  **SecY**  and either E. coli SecE or E. coli  **SecG**  were present.  Ref: 10559168 J Bacteriol, 1999
- SecD, SecF,  **SecG** , and  **SecY**  were decreased 4 6 fold.  Ref: 9153216 J Biol Chem, 1997
- These prokaryotic eukaryotic proteins include 1  **SecY**  Sec61alpha, 2 SecE Sec61gamma, 3  **SecG**  Sec61beta, 4 Ffh SRP54 and 5 FtsY SRP receptor subunit alpha.  Ref: 12507766 Biochim Biophys Acta, 2003
- ... the  **SecY**  SecE complex without  **SecG**  dissociated at a slightly lower temperature 20 23 degrees C.  Ref: 9180258 FEBS Lett, 1997
- We used hexahistidine tagged SecE and  **SecY**  to study how the core subunits  **SecY** , SecE and  **SecG**  of Escherichia coli protein translocase interact with each other.  Ref: 9180258 FEBS Lett, 1997
- Translocation occurs at a multisubunit membrane bound enzyme termed translocase, which consists of a peripheral preprotein binding site and an ATPase domain termed SecA, a core heterotrimeric integral membrane protein complex with  **SecY** , SecE and  **SecG**  as subunits, and an accessory integral membrane protein complex containing SecD and SecF.  Ref: 10066476 Curr Opin Microbiol, 1998
- However, the  **SecG**  dependent stabilization of  **SecY**  was less potent than the SecE dependent stabilization.  Ref: 8526914 Biochem Biophys Res Commun, 1995
- Although His6 SecE, expressed in wild type cells, brought down both  **SecY**  and  **SecG** , neither of them was brought down when the same protein was expressed in the secY24 mutant cells.  Ref: 9180258 FEBS Lett, 1997
- Subunit interactions in the Escherichia coli protein translocase SecE and  **SecG**  associate independently with  **SecY** .  Ref: 9180258 FEBS Lett, 1997
- SecYEG, a complex of integral membrane proteins  **SecY** , SecE, and  **SecG**  in the cytoplasmic membrane, constitutes a pathway for polypeptide movement.  Ref: 12812064 Yi Chuan Xue Bao, 2003
- Membrane vesicles prepared from these cells, when grown at the nonpermissive temperature, contained about 5%  **SecY**  and similarly low levels of  **SecG** .  Ref: 9393703 J Bacteriol, 1997
- Deletion of the N terminal region of SecE also rendered the SecE  **SecY**   **SecG**  complex unstable.  Ref: 11129584 Biosci Biotechnol Biochem, 2000
- We now report that both prlA and prlG mutations weaken the associations between the  **SecY** , SecE and  **SecG**  subunits of the translocase.  Ref: 10369667 EMBO J, 1999
- **SecY** , SecE and  **SecG**  form a membrane part of the protein translocation machinery.  Ref: 8526914 Biochem Biophys Res Commun, 1995
- Upon release of the SRP at the membrane, the targeted nascent proteins insert into a translocon that contains at least SecA,  **SecY**  and  **SecG** .  Ref: 9564033 EMBO J, 1998
- Identification of a  **SecY**   **SecG**  Interface.  Ref: 12809499 Biochemistry, 2003
- These results confirm our previous conclusion that the secY24 alteration impairs the  **SecY**  SecE interaction, and demonstrate that  **SecY**  and  **SecG**  can form a complex that does not contain SecE.  Ref: 9180258 FEBS Lett, 1997
- **SecY**   **SecY**  and  **SecY**   **SecG**  contacts revealed by site specific crosslinking.  Ref: 12220653 FEBS Lett, 2002
- These data establish that  **SecY** , SecE, and  **SecG**  constitute the integral membrane domain of preprotein translocase.  Ref: 7650029 J Biol Chem, 1995
- Escherichia coli preprotein translocase contains a membrane embedded trimeric complex of  **SecY** , SecE and  **SecG**  SecYEG and the peripheral SecA protein.  Ref: 9184221 EMBO J, 1997
- On the other hand, the ATPase activity of SecA, which was dependent on proOmpA and  **SecY** .SecE containing proteoliposomes, was significantly enhanced when the proteoliposomes contained  **SecG** .  Ref: 8089132 J Biol Chem, 1994
- Reconstitution of an efficient protein translocation machinery comprising SecA and the three membrane proteins,  **SecY** , SecE, and  **SecG**  p12.  Ref: 8089132 J Biol Chem, 1994
- The levels of  **SecY**  and  **SecG**  in the secEcsE501 mutant, which expresses SecE at a decreased level and is sensitive to low temperature, increased upon the expression of functional SecE derivatives, irrespective of the site of mutation, suggesting that the levels of  **SecY**  and  **SecG**  are co operatively determined by the level of functional, but not non functional, SecE.  Ref: 8827448 J Biochem Tokyo , 1996
- A chimeric SecE possessing the cytoplasmic region of the E. coli SecE and the following region of the Bacillus subtilis SecE was able to form the translocation machinery together with SecA,  **SecY** , and  **SecG** .  Ref: 8827448 J Biochem Tokyo , 1996
- **SecY** , SecE and  **SecG**  form a heterotrimer, which functions as a protein translocation channel in Escherichia coli.  Ref: 12655407 Mol Genet Genomics, 2003
- To systematically determine the functional exchangeability of translocase subunits, all of the relevant combinations of the E. coli and B. subtilis  **secY** , secE, and  **secG**  genes were expressed in E. coli.  Ref: 10559168 J Bacteriol, 1999
- Preprotein translocation in Escherichia coli is mediated by the translocase with SecA as peripheral ATPase and  **SecY** , SecE, and  **SecG**  as membrane domain.  Ref: 9425040 Biochemistry, 1998
- Precursor protein translocation across the Escherichia coli inner membrane is mediated by the translocase, which is composed of a heterotrimeric integral membrane protein complex with  **SecY** , SecE, and  **SecG**  as subunits and peripherally bound SecA.  Ref: 9294424 J Bacteriol, 1997
- However, despite such a dramatic reduction in  **SecY**  and  **SecG**  levels, these membranes exhibited 50 to 70% of the wild type translocation activity, including the processing of the signal peptide, of OmpA precursor proOmpA.  Ref: 9393703 J Bacteriol, 1997
- YidC could not be integrated in the absence of  **SecY** , SecE, or  **SecG** , suggesting that YidC, in contrast to its mitochondrial orthologue Oxa1p, cannot engage a SecYEG independent protein conducting channel.  Ref: 11777926 J Biol Chem, 2002
- Integral membrane components  **SecY** , SecE, and  **SecG**  of protein translocase form a complex in the Escherichia coli plasma membrane.  Ref: 12809499 Biochemistry, 2003
- Experiments using spheroplasts revealed the physical proximity of P2  **SecY**  and the C terminal periplasmic region of  **SecG** .  Ref: 12809499 Biochemistry, 2003
- Bacterial protein translocation is mediated by translocase, a multisubunit membrane protein complex that consists of a peripheral ATPase SecA and a preprotein conducting channel with  **SecY** , SecE, and  **SecG**  as subunits.  Ref: 10559168 J Bacteriol, 1999
- We have now placed the  **secY** , secE, and  **secG**  genes under the control of an arabinose inducible promoter on a multicopy plasmid.  Ref: 7650029 J Biol Chem, 1995
- Escherichia coli preprotein translocase comprises a membrane embedded trimeric complex of  **SecY** , SecE and  **SecG** .  Ref: 11867527 EMBO J, 2002
- Experiments using inverted membrane vesicles revealed specific cross linkings between a cysteine residue placed in the C2 or C3 domain of  **SecY**  and the cytosolic lysine Lys26 near the first transmembrane segment of  **SecG** .  Ref: 12809499 Biochemistry, 2003
- Detailed kinetic analyses indicated that the activity of proteoliposomes was dependent on the amount of the  **SecY**  SecE complex when  **SecG**  was absent and the amount of the  **SecY** .SecE. **SecG**  complex when the proteoliposomes contained  **SecG** .  Ref: 8089132 J Biol Chem, 1994
- Overproduction of the integral subunits of the translocase, the  **SecY** , SecE and  **SecG**  polypeptides, results in an increased level of membrane bound SecA.  Ref: 8951810 Mol Microbiol, 1996
- While SecE and  **SecY**  are generally accepted as essential components of translocase, the role of  **SecG**  is more ambiguous.  Ref: 10732681 Mol Gen Genet, 2000
- **SecY** , SecE and  **SecG**  form the membrane embedded core complex of the Escherichia coli protein export apparatus.  Ref: 10732681 Mol Gen Genet, 2000
- We conclude that each of SecE and  **SecG**  independently binds to  **SecY** , the central subunit of protein translocase, although the trimeric complex is more stable than the binary complexes.  Ref: 9180258 FEBS Lett, 1997
